# TreeToReads - a pipeline for simulating raw reads from phylogenies

**DOI:** 10.1186/s12859-017-1592-1

**Published:** 2017-03-20

**Authors:** Emily Jane McTavish, James Pettengill, Steven Davis, Hugh Rand, Errol Strain, Marc Allard, Ruth E. Timme

**Affiliations:** 1 University of California, Merced, Merced, CA USA; 2 University of Kansas, Lawrence, RS USA; 3 Center for Food Safety and Nutrition, Food and Drug Administration, College Park, MD USA

**Keywords:** Genomics, Phylogenetics, Simulation

## Abstract

**Background:**

Using phylogenomic analysis tools for tracking pathogens has become standard practice in academia, public health agencies, and large industries. Using the same raw read genomic data as input, there are several different approaches being used to infer phylogenetic tree. These include many different SNP pipelines, wgMLST approaches, k-mer algorithms, whole genome alignment and others; each of these has advantages and disadvantages, some have been extensively validated, some are faster, some have higher resolution. A few of these analysis approaches are well-integrated into the regulatory process of US Federal agencies (e.g. the FDA’s SNP pipeline for tracking foodborne pathogens). However, despite extensive validation on benchmark datasets and comparison with other pipelines, we lack methods for fully exploring the effects of multiple parameter values in each pipeline that can potentially have an effect on whether the correct phylogenetic tree is recovered.

**Results:**

To resolve this problem, we offer a program, TreeToReads, which can generate raw read data from mutated genomes simulated under a known phylogeny. This simulation pipeline allows direct comparisons of simulated and observed data in a controlled environment. At each step of these simulations, researchers can vary parameters of interest (e.g., input tree topology, amount of sequence divergence, rate of indels, read coverage, distance of reference genome, etc) to assess the effects of various parameter values on correctly calling SNPs and reconstructing an accurate tree.

**Conclusions:**

Such critical assessments of the accuracy and robustness of analytical pipelines are essential to progress in both research and applied settings.

## Background

Whole genome sequencing (WGS) now allows researchers to trace ancestry among samples that differ by only a few mutations. Phylogenetic trees inferred from WGS data are a valuable tool for tracing the ancestry of closely related lineages in bacterial pathogen outbreaks. However, these estimates of shared ancestry may rely on only a handful of data points. When the resulting phylogenetic trees are used by public health agencies to make public health decisions, such as to define the scope of foodborne outbreaks [1], to identify the source of these outbreaks [2–4] and where appropriate to follow-up with regulatory or legal actions, it is particularly important to ensure that the WGS analysis methods used are validated.

One potential source of error is biases in which variable sites (single nucleotide polymorphisms, or SNPs) are detected from analysis of the sequencing read data. SNP ascertainment biases can be caused by various factors. These biases can be affected by analysis parameters, such as using missing data cutoffs [5] or read filtering artifacts [6]. Read mapping issues due to choice of reference genome [7] and different mapping algorithms [8] can also result in biases in which variable sites are detected. Phylogenetic error can be exacerbated by interaction among dataset biases and analytic choices; for example, using a model of evolution developed for sequence data on a panel of exclusively variable sites [9], or choosing an inappropriate model of evolution [10].

Despite the sheer quantities of genomic data, it is possible that these types of biases could affect phylogenetic conclusions. If these errors are systematic, analyses can converge to support an incorrect result with high bootstrap confidence. In order to adopt data analysis pipelines for the regulatory environment it is necessary to understand potential biases in sequence analysis pipelines and validate their use. Simulations are a useful approach to investigating potential biases. Without validated in silico modeling, scientists have to rely on benchmark datasets where the truth can never be truly known.

To help solve this problem, we present TreeToReads, a software tool for simulating realistic patterns of sequence variation across phylogenies. This pipeline may be used to assess the robustness of evolutionary inferences from whole genome data against potential biases inherent in data collection and analysis pipelines. Two key aspects of TreeToReads differentiate it from existing simulation alternatives. First, the variable sites follow a user-specified phylogeny, resulting in more realistic evolutionary patterns. Second, those variable sites are placed in the context of an observed or ‘anchor genome’, which is represented as the tip on that phylogeny. Together, this combination allows researchers to make direct comparisons of WGS read mapping between their observed and simulated reads, and thus makes TreeToReads appropriate for testing the effects of distance to a reference genome on phylogenetic inference from genomic data. That anchor genome can be used as a reference genome for read mapping, or mapping can be tested against other empirically-observed genomes. In the case of empirically-observed genomes, what separates the reference data from the simulated data constitutes real evolutionary history. Making direct read mapping comparisons to assess reference genome effects cannot be done using alternative simulation software.

## Implementation

**Fig. 1:**
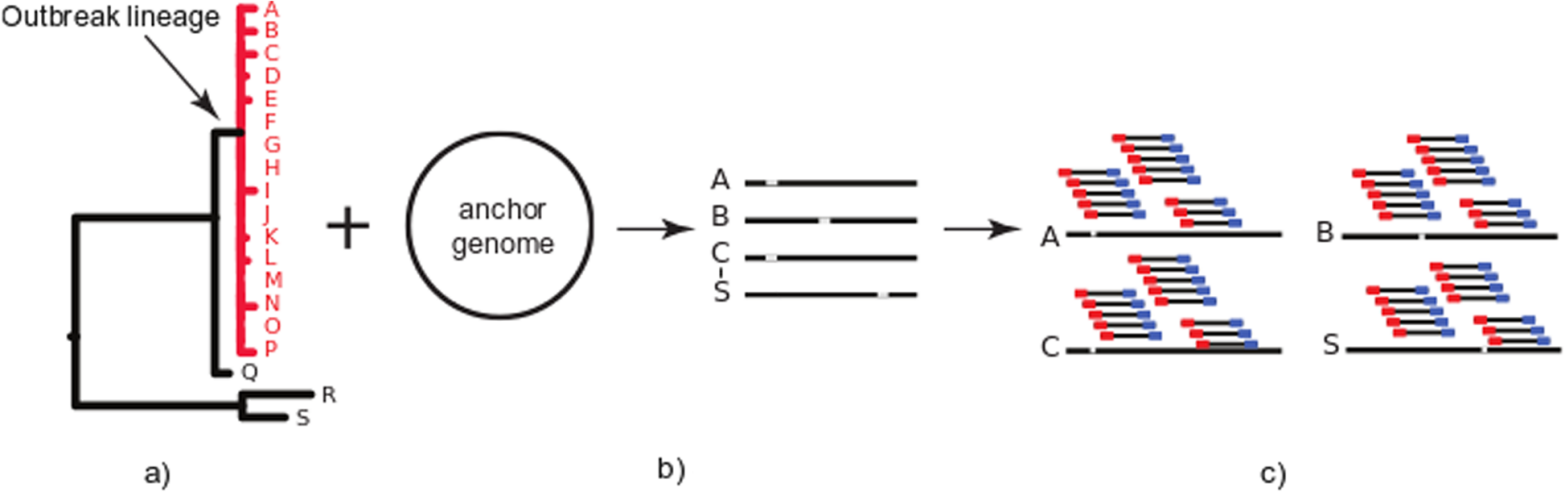
Schematic of the TreeToReads procedure. **a** Input Newick tree file and background/anchor genome. **b** Simulate mutations across taxa according to defined set of parameters. **c** Simulate raw reads (fastq files)

The TreeToReads pipeline generates short read data from genomes simulated along an input phylogeny (Fig. 1). The software is written in Python and requires a configuration file and two input files - a phylogeny with branch lengths and a FASTA formatted genome sequence that serves as the anchor genome. The user specifies parameter settings (e.g., number of variable sites to simulate and nucleotide substitution model parameters) in a configuration file. The branch lengths of the user-provided phylogeny determine the relative number of mutations on each branch and the probability that a single site is affected by multiple mutational events. Base frequencies are calculated from the composition of the anchor genome. To account for the ascertainment bias inherent in estimating phylogenies from panels of variable sites, the total number of variable sites is determined by a user input parameter, not the branch lengths. The pipeline uses Seq-Gen [11] to simulate variable sites along the input phylogeny under the selected model of evolution. The locations of mutations in the genome are either drawn from a uniform distribution, or clustered according to parameters of an exponential distribution specified in the configuration file. For each mutation site, an alignment column is drawn from the Seq-Gen simulation which has the correct base at the anchor genome, and at least one alternate base at another tip, and the simulated bases are assigned to each tip. Therefore the pattern of evolution at the site fits the phylogeny and model of evolution, but the anchor genome remains unchanged. This procedure requires the use of a single model of evolution across the genome. Insertions and deletions can be included in the simulation, using INDELible [12]. which requires an indel rate parameter and distribution. The indel rate is scaled per expected substation per site, and therefore the number of insertions and deletion scales with the simulated number of SNPs. All insertions are with respect to the reference genome, so the reference remains unchanged, although there are apparent deletions, caused by insertions in other lineages. The inserted sequences are generated from scratch according to the parameters of the evolutionary model. The pipeline creates an output folder for each tip in the tree that contains the simulated genomes (FASTA files). A user-specified tip will consist of the input anchor genome without any mutations. Using these simulated genomes, TreeToReads calls the read simulation software, ART, [13] to generate Illumina MiSeq paired-end reads. The user can apply a default sequence error model, or use the configuration file to specify an error model generated for observed data. TreeToReads currently supports automated generation of Illumina paired end reads. For other read types, the simulated genome files may be used outside of TreeToReads with any ART parameter configuration. Alternatively, if RAD-seq like data are desired other raw-read generators such as SimRAD [14] can be used on the simulated genomes from TreeToReads. If ART is invoked in TreeToReads the program will output a folder labeled ‘fastq’ containing directories labeled with the names of each tip from the simulation tree, in which the simulated reads are deposited in.fastq.gz formats. A vcf file with the location and nucleotide state of each mutation as mapped to the anchor genome is also output.

## Results and discussion

To validate the performance of TreeToReads, we performed 5 replicate analyses under each of two case study conditions then compared the inferences to the simulation parameters. In the first case study we also assessed the effects of distance to reference genome on inference. All validation configuration files, input files, analysis scripts and output files are available with the software on GitHub https://github.com/snacktavish/TreeToReads.

The first case study used whole genome read data from ten *Salmonella enterica* sequences associated with a 2010 outbreak, and the observed phylogeny inferred from those data [2]. We used a completed *Salmonella enterica* genome (CFSAN000189, GenBank: CP006053.1) as the anchor sequence, and simulated 200 variable sites under the generalized time reversible (GTR) model at an average coverage of 20 reads per site. Based on the locations of variable sites in the observed sequence data, the distances between locations for 20% of the variable sites were drawn from an exponential distribution with a 125bp mean. The locations for the rest of the variable sites were drawn from a uniform distribution across the genome. The read error profile was based on the observed outbreak sequence data.

For the second case study we simulated data using the reference genome and phylogeny from a *Listeria* outbreak. The tree and associated metadata are available in the Open Tree of Life data store (tree.opentreeoflife.org/curator/study/view/ot_301) [15]). For the validation tests we pruned out multiple replicates of clonal lineages, to create a resolved tree. We used a completed genome from the outbreak, *Listeria monocytogenes* (CFSAN023463, GenBank NZ_CP012021.1) as the anchor sequence. 500 variable sites were distributed uniformly across the genome. Insertions and deletions were simulated under the Lavalette distribution, with a = 1.7 and a maximum size of 541 base pairs [12]. The insertion and deletion rates were equal and 0.1 times the nucleotide substitution rate. To demonstrate that sequence data can be simulated without empirical data in hand, for this case study the sequencing error rate was drawn from the defaults provided with ART. Reads were simulated at an average coverage of 40 reads per site [13].

For each replicate we analyzed the resulting short read datasets using the SNP pipeline from the Food and Drug Administration, Center for Food Safety And Nutrition (FDA CFSAN) to identify SNPs in each simulated dataset [16]. The CFSAN SNP Pipeline uses reference-based alignments to create a matrix of SNPs for a given set of samples. We used the anchor genomes as the reference genomes for both case studies. For the first case study, *Salmonella*, we also ran these analyses using a distant reference genome, *Salmonella enterica* Typhimurium str. LT2 (GenBank AE006468.2). This reference differs from the anchor genome at around 0.7% percent of sites; approximately 36,490 nucleotide sites, across the 4,730,612 base pair Bareilly outbreak anchor genome (CFSAN000189), although the exact count depends on alignment choices. Using the called SNPs we inferred the maximum likelihood phylogeny for each data set using RAxML [17]. For the analyses using closely related reference genomes we applied the ASC GTRGAMMA model with a Lewis ascertainment bias correction, which corrects for including only variable sites in the analysis [9]. For the analyses using the distant reference genome many called SNPs were actually fixed differences from the reference and not variable within the alignment. We used the GTRGAMMA model with no ascertainment bias correction for phylogenetic analyses on these data.

We then compared the inferences from the data simulated using TreeToReads to the input parameters for the topology, number of variable sites, base frequencies, and GTR model of evolution. We compared the inferred topology to the input topology using Robinson–Foulds (RF) distances [18] - the symmetric difference in partitions between the input and inferred trees using Dendropy [19]. For the *Listeria* example we also compared the indel locations in the simulated alignment to that generated by INDELible [12]. We do not independently test the correctness of the results of INDELible, but the indel locations were in all cases were identical to those output by INDELible.

**Fig. 2:**
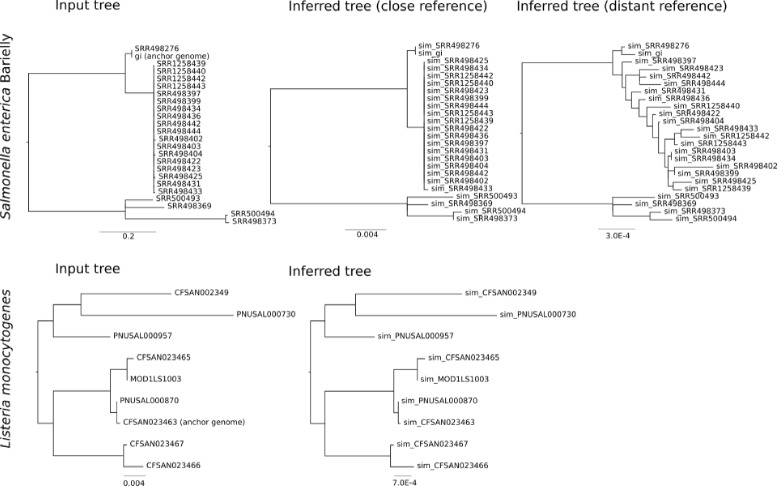
Input and inferred trees for two case studies, *Salmonella enterica* Bareilly and *Listeria monocytogenes*. Variable sites called for *Salmonella enterica* Bareilly by mapping reads to the anchor genome as a reference (close reference), and to a reference genome outside of the sampled tree (distant reference). *Listeria monocytogenes* reads were mapped to the anchor genome

The input parameters and the mean and standard deviation for inferred parameters across 5 replicates are reported in Table 1. A representative tree inferred in a single replicate for each parameter set is shown in Fig. 2. In all replicates the simulated number of variable sites was exactly the input number of SNPs, and using closely related reference genomes resulted in correct inferences of nearly all simulated SNPs. On average the SNP caller misses 1% of variable sites, and there were no false positive SNP calls when using closely related references. Using a distant reference genome in the *Salmonella* case study resulted in inference of many ‘variable’ sites (∼38,229) which were actually fixed differences between the reference and the anchor genome. Including these sites which are invariant within the sampled lineages should not be problematic for inference. However, using this distant reference genome also resulted in inference of at least 55 false positive variable sites within the sampled lineages. These false positives are likely due to mis-mapping of reads onto the divergent reference genome, creating the appearance of variable sites. These incorrect SNP calls resulted in incorrect inferences of phylogenetic structure within the clonal outbreak group (Fig. 2). Nonetheless, the key branch, leading to the outbreak lineages, was reconstructed correctly in every replicate, even when reads were mapped to the distant reference. Mapping to the close reference, the resolution of very short branches among the clonal species was effectively arbitrary, as would be expected by inferring bifurcating relationships at a hard polytomy. No inferred topology exactly matched that of the input tree for the *Salmonella* case study. As RF distance treats bifurcations equally without respect to branch length, RF distances between the input and inferred trees are high across all of the *Salmonella* replicates. In the *Listeria* case study every inferred topology matched the input topology exactly. We do not directly compare branch lengths, as in analyses of exclusively variable sites, branch lengths are challenging to estimate even under an ascertainment bias correction. Nonetheless, when SNPs were called using a closely related reference genome, the relative branch lengths of the inferred tree reflect those of the input tree.

**Table 1:** Simulation parameter values and mean of inferred parameter values across 5 simulation and inference replicates

		Case Study 1 – *Salmonella enterica* Bareilly			
		Input	Inferred		
			Close ref	Far ref	
Number of SNPs		100	98.6 (1.4)	38,384 (mutations)	
				154 (3) variable sites	
RF distance		-	33 (1)	33 (1)	
Outbreak clade monophyletic		-	5/5	5/5	
Base frequencies	A	0.238787	0.24 (0.03)	0.25 (0.0)	
	C	0.261361	0.26 (0.03)	0.25 (0.0)	
	G	0.261132	0.24 (0.03)	0.25 (0.0)	
	T	0.238720	0.27 (0.03)	0.24 (0.0005)	
GTR rate Matrix	ac	0.4	0.1 (0.7)	1.5 (0.6)	
	ag	T 3.0	91 (160)	5.4 (2.0)	
	at	0.5	21 (39)	1.0 (0.6)	
	cg	0.1	0.1 (0.2)	1.5 (0.7)	
	ct	4.4	66 (112)	5.3 (1.9)	
	gt	1	1.0 (0.0)	1.0 (0.0)	
Coverage		20	17.04 (0)	15.45 (0)	
		Case Study 2 – *Listeria monocytogenes*			
		Input	Inferred		
Number of SNPs		500	494.8 (1.6)		
RF distance		-	0.0 (0)		
Base frequencies	A	0.311521	0.30 (0.01)		
	C	0.190709	0.20 (0.01)		
	G	0.189125	0.20 (0.007)		
	T	0.308645	0.29 (0.007)		
GTR rate Matrix	ac	1.2070	1.1 (0.1)		
	ag	5.9306	5.3 (0.8)		
	at	1.7425	1.8 (0.2)		
	cg	0.4610	0.3 (0.2)		
	ct	5.1238	5.1 (0.7)		
	gt	1	1.0 (0.0)		
Coverage		40	37.49 (0)		
Difference from INDELible gap distribution		0	0.0 (0)		

Standard deviations of parameter values shown in parentheses

In the *Salmonella* case study, where there were only 100 variable sites, inferences of base frequencies and GTR rate parameters did not match the simulated conditions (Table 1). Evolutionary rate parameters are difficult to estimate when the observed number of mutations in a single category are rare, and some of the relative rate parameters made certain rate categories (e.g. ac, cg) very rare, resulting in high variance across replicates in inferred rates. However, in the *Listeria* case study, where 400 variable sites were simulated, providing sufficient data to accurately infer rates, the simulated input parameters were within one standard deviation of the mean inferences of parameter values (Table 1).

These examples demonstrate that TreeToReads is effectively simulating data under complex evolutionary scenarios. In straightforward analysis cases, the inferred parameters match almost exactly the input parameters, although this is subject to data set size, and ability to resolve relationships of interest. Although many software tools can simulate sequence data, no existing tools combine phylogenetic relationships with observed genomic sequences. TreeToReads is designed to test the effects of multiple parameters on phylogenetic inference and provides a pipeline to simulate next-gen sequencing reads from a phylogenetic tree using an observed error model. Using an anchor genome as a tip in the simulated tree means that simulated and empirical data can be mapped to the same reference genome, providing direct comparisons of inferences. In addition, the reference genome does not need to be the anchor genome on which the simulations are based. If a different reference is used, as in the *Salmonella* analyses with a distance reference, the biological evolution separating the anchor genome from the reference genome includes real evolutionary processes affecting read mapping to genomes in a testable framework. This provides a greater realism than can be provided by even the most complex available models for simulating sequence evolution. Alternatively, the user can use the simulated data to test reference-free methods for phylogenetic inference, such as multi locus sequence typing (MLST) [20], or the k-mer based method kSNP [21].

Seq-Gen [11], used to generate the variable sites in the TreeToReads pipeline, uses a full GTR model of evolution with parameters specified by the user. However, on its own, Seq-Gen generates random sequences based on the model of evolution and therefore does not incorporate observed genomic context. Consequently, reads from Seq-Gen-simulated genomes cannot be mapped to observed reference genomes. This is also true for other simulators of more complex evolutionary processes, such as SWGE [22]. Other sequence simulation software, such as ALF [23] and Indel Seq-gen [24], simulate evolution forward in time, starting from an input genome representing the root of the phylogeny. However, in empirical data the reference genome is not an ancestor - it is always a present day relative. Anchoring an observed genome to a tip in a tree using TreeToReads allows us to test choices about selection of reference genomes in a way that is directly comparable to empirical data. SWGE and ALF include complex evolutionary processes not simulated in TreeToReads, such as recombination across lineages and heterogeneity of the evolutionary model across the genome. While recombination and horizontal gene transfer (HGT) can be important processes, the expectations are challenging to parameterize, as even a few HGT events can lead to phylogenetic networks generating extremely complex ancestral recombination graphs [25]. In order to simulate shifts in relationships along chromosomes it is possible to independently input into TreeToReads different tree topologies for different genomic regions, and then combine the reads for these simulations and attempt inference. TreeToReads is designed for testing the robustness of pipelines for estimating bifurcating relationships from next generation sequencing read data. While inference of branch lengths can be biased by recombination [26], as well as by selection and use of exclusively variable sites to infer phylogenies [17], inference of topology is often robust to recombination [26].

## Conclusions

Existing genomic simulation software packages cannot provide a phylogenetic perspective in simulation testing of assembly and alignment tools. TreeToReads allows researchers to test the joint effects of multiple parameter values on the ability of any analysis pipeline to recover the signal and infer the correct tree. Simulating data that spans these parameters can validate methods for reconstructing phylogenies directly from short-read data, which is especially useful for public health agencies tracking emerging pathogens.

## Availability and requirements

**Project name:** TreeToReads

**Project home page:** https://github.com/snacktavish/TreeToReads

**Operating system(s):** Linux, OSX

**Programming language:** Python

**Other requirements:** Dependencies, Seq-Gen [11], Art [13], Dendropy [19]

**Licence:** This project constitutes a work of the United States Government and is not subject to domestic copyright protection under 17 USC *§* 105.

## Abbreviations

SNP: Single nucleotide polymorphism
WGS: Whole genome sequence
